# Impacts of inter-trial interval duration on a computational model of sign-tracking vs. goal-tracking behaviour

**DOI:** 10.1007/s00213-019-05323-y

**Published:** 2019-07-31

**Authors:** François Cinotti, Alain R. Marchand, Matthew R. Roesch, Benoît Girard, Mehdi Khamassi

**Affiliations:** 1 CNRS, Institut des Systèmes Intelligents et de Robotique (ISIR), Équipe AMAC, Sorbonne Université, 4 Place Jussieu, 75252 Paris cedex 05, France; 2 CNRS, Institut de Neurosciences Cognitives et Intégratives d’Aquitaine (INCIA, UMR5287), Bordeaux, France; 3 INCIA, Université de Bordeaux, Bordeaux, France; 4 Department of Psychology, University of Maryland, College Park, MD USA; 5 Neuroscience and Cognitive Science Program, University of Maryland, College Park, MD USA

**Keywords:** Sign-tracking, Goal-tracking, Reinforcement learning

## Abstract

In the context of Pavlovian conditioning, two types of behaviour may emerge within the population (Flagel et al. Nature, 469(7328): 53–57, 2011). Animals may choose to engage either with the conditioned stimulus (CS), a behaviour known as sign-tracking (ST) which is sensitive to dopamine inhibition for its acquisition, or with the food cup in which the reward or unconditioned stimulus (US) will eventually be delivered, a behaviour known as goal-tracking (GT) which is dependent on dopamine for its expression only. Previous work by Lesaint et al. (PLoS Comput Biol, 10(2), 2014) offered a computational explanation for these phenomena and led to the prediction that varying the duration of the inter-trial interval (ITI) would change the relative ST-GT proportion in the population as well as phasic dopamine responses. A recent study verified this prediction, but also found a rich variance of ST and GT behaviours within the trial which goes beyond the original computational model. In this paper, we provide a computational perspective on these novel results.

**Electronic supplementary material:**

The online version of this article (10.1007/s00213-019-05323-y) contains supplementary material, which is available to authorized users.

## Introduction

Individual differences in response to conditioned stimuli (CSs) have elicited much interest in the recent years as a model of differential susceptibility to drug addiction (Saunders and Robinson 2013). In a Pavlovian appetitive task where a CS cue invariably predicts the occurrence of a biologically relevant event (US) such as a food reward, animals could be expected to direct their response towards the locus of food delivery (goal-tracking or GT), but a sub-population of subjects focus on the cue itself instead, as if it had acquired incentive properties similar to those of the reward (Meyer et al. 2012). The latter form of behaviour, termed sign-tracking (ST), has been reported in several species, including pigeons (Jenkins and Moore 1973) as well as rats (Robinson and Flagel 2009) in a Pavlovian task where the CS is the presentation of an inactive lever. Sign-tracking is thought to be a stable trait of individuals (sign-trackers, STs) that are more prone to display an automatic behaviour towards reward-predicting cues, in the sense that the same animals may be less sensitive to extinction of conditioning (Ahrens et al. 2016) or to devaluation of the reward (Morrison et al. 2015; Nasser et al. 2015; Patitucci et al. 2016) than individuals exhibiting goal-tracking (known as goal-trackers, or GTs) although this has recently been contested by Derman et al. (2018).

Many interpretations of sign-tracking have been proposed, including potentiation of orienting responses dependent on CS-US pairing (Holland 1980), or instrumental conditioning due to adventitious response-reinforcer associations (Davey et al. 1981). Nevertheless, sign-tracking has been more recently construed as the acquisition of incentive motivation by the conditioned cue (Berridge 2012). One of the arguments in favour of this interpretation is that the acquisition of sign-tracking, but not goal-tracking, is dependent upon dopaminergic signalling, specifically in the core of the nucleus accumbens (Saunders and Robinson 2012; Scülfort et al. 2016; Fraser and Janak 2017; Lee et al. 2018). Moreover, goal-tracking and sign-tracking are associated with distinctive patterns of dopamine signalling in this brain region (Flagel et al. 2011).

Several models have been proposed to explain the motivational role of dopamine during acquisition and/or expression of sign-tracking (Zhang et al. 2009; Kaveri and Nakahara 2014; Anselme 2015), but few of them account for individual differences in the form of conditioned responses, namely the existence of separate populations of STs and GTs (Kaveri and Nakahara 2014). One of the hypotheses proposed is the existence of parallel learning processes relying on different error signals and providing different degrees of behavioural flexibility. In the present computational study, we examine the ability of one such model (Lesaint et al. 2014; Lesaint et al. 2014; Lesaint et al. 2015) to replicate new experimental findings (Lee et al. 2018) from an experiment specially designed to test predictions the model had made (Lesaint et al. 2015).

## Description of the original experiments and of the original FMF-MB model

The model proposed by Lesaint et al. (2014) was aimed at explaining a Pavlovian conditioning experiment designed by Flagel et al. (2009) which consisted in the 8s-presentation of a retractable lever as a CS which would be immediately followed by the delivery of a food pellet (the unconditioned stimulus, or US) in a nearby magazine. This training took place over several sessions of fifty trials each, and the researchers recorded behavioural measurements during the CS period such as the number of times the animal touched the lever or entered the food port, the latencies between lever appearance and the first contact with either of these stimuli and the probability of interacting with them. The main results of this first paper was the gradual development of different types of responses within the population, with one sub-group known as sign-trackers increasingly interacting with the lever, and another sub-group called goal-trackers who preferred to go straight towards the magazine where the food would be delivered later. Following this first paper, Flagel et al. (2011) investigated the role of dopamine in these types of behaviours by training rats selectively bred for their sign-tracking or goal-tracking tendencies on this task under pharmacological inhibition of dopamine receptors, before testing the rats without inhibition. In this study, they found that dopamine was necessary for the acquisition of sign-tracking behaviour as it was still reduced in the test session, but this was not the case for goal-tracking which was thus proved to be dependent on dopamine for its expression only.

**Fig. 1:**
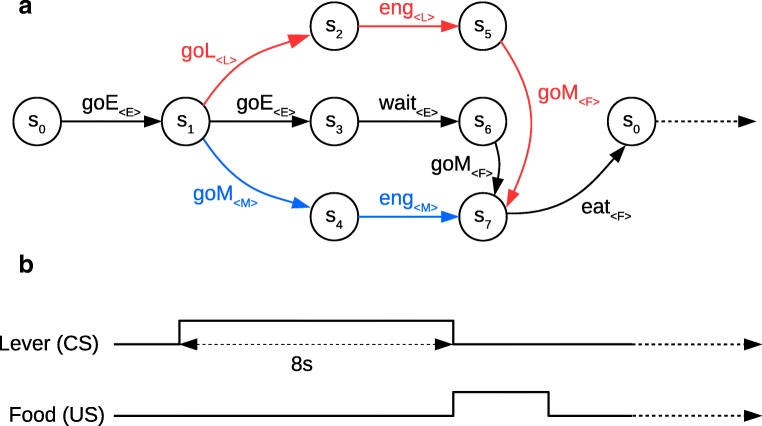
**a** MDP representation of a single trial from the original experiment by Flagel et al. (2009) adapted from Lesaint et al. (2014). There are six possible actions leading deterministically from one state to the next: exploring the environment (goE), approaching the lever (goL), approaching the magazine (goM), waiting, engaging with the closest stimulus, and eating the reward. Each of these actions focuses on a specific feature indicated in brackets: the environment (E), the lever (L), the magazine (M), and the food (F). These are the features used by the FMF learning component. The red path corresponds to sign-tracking behaviour and the blue path to goal-tracking behaviour. **b** Corresponding timeline of lever and food appearances

**Fig. 2:**
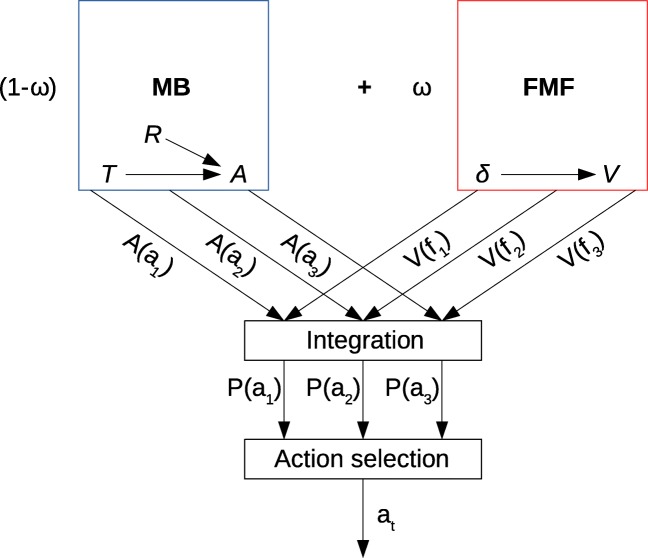
Schematic representation of the FMF-MB decision-making model adapted from Lesaint et al. (2014). The model combines a Model-Based learning system which learns the structure of the MDP and then calculates the relative advantage of each action in a given state, with a Feature-Model-Free system which attributes a value to different features of the environment which is generalized across states (e.g. the same value of the magazine is used in states 1 and 4). The advantage function and value function are weighted by *ω*, their relative importance determining the sign- vs goal-tracking tendency of the individual and then passed to the action selection mechanism modelled by a softmax function

These are the main results used by Lesaint et al. (2014) to devise their model of this behaviour. Computationally, the structure of each trial of the task is represented by a Markovian Decision Process (MDP) consisting of seven different states (Fig. 1) defined by the environmental conditions, such as the presence of the lever or of the food, and the current position of the animal (close to the food tray or to the lever). There are six different actions (explore the environment or goE, approach the lever or goL, approach the magazine or goM, wait, engage the closest stimulus and eat the reward), and state transitions given a selected action are deterministic, e.g. if an animal in state 1 chooses the action goL, it will always land in state 2. The decision-making model itself (Fig. 2) consists of a Model-Based (MB) and of a Feature-Model-Free (FMF) learning system which output respectively an advantage function and a value function for all possible actions in the current state. These two functions are then combined into a weighted mean defined by the *ω* parameter representing how much each system contributes to final behaviour. A high *ω* gives more importance to the value function computed by the FMF system, while a low *ω* favours the MB system. Finally, the weighted averages are given to a softmax function representing the action selection mechanism (Fig. 2).

An original aspect of the FMF system compared with other reinforcement learning algorithms is that it assigns a value representing future expected reward not to states or actions but to the feature which the different actions are focused on. This allows for a generalization of values between different states, e.g. when the animal goes towards the magazine in state 1 or engages the magazine in state 4, it is the same feature value *V* (*M*) which is called upon. The feature which the animal’s current action is aimed at is defined by the feature-function f (see Table 1). After each action, observing the delivery of the reward r (equal to 1 or 0 if delivery takes place or not, respectively) and the new state *s*_*t*+ 1_, the animal updates the value the last action was aimed at using standard temporal-difference learning rules. Firstly, it computes a reward prediction error (RPE), *δ*, which is the difference between the observed outcome, which consists in the presence or absence of a reward and the discounted value of the next best possible feature, and the expected outcome, i.e. the previous estimate of the value of the chosen feature:

1 $$ \delta = r + \gamma \max_{j}(V(f(s_{t+1}, a_{j}))) - V(f(s_{t},a_{t}))  $$

with *γ* the discounting factor bounded between 0 and 1. This RPE is then integrated in the current estimate of the value of the chosen feature:

2 $$ V(f(s_{t}, a_{t})) \leftarrow V(f(s_{t}, a_{t})) + \alpha \delta  $$

with *α* the learning rate bounded between 0 and 1. The higher *α* is, the faster an agent can learn but at the cost of increasing unstability which can be very detrimental in a noisy environment (which is not our case as rewards and transitions are all deterministic).

**Table 1:** Feature function mapping state-action pairs to features as required for the feature model-free learning system. For the state-action pair s_7_-eat, we used either food as originally designed in Lesaint et al. (2014) or the magazine when trying to explain dopaminergic activity, as explained in the main text

States	*s*_0_	*s*_1_	*s*_1_	*s*_1_	*s*_2_	*s*_3_	*s*_4_	*s*_5_	*s*_6_	*s*_7_
Actions	goE	goL	goE	goM	eng	wait	eng	goM	goM	eat
*f*(*s*,*a*)	*E*	*L*	*E*	*M*	*L*	*E*	*M*	*F*	*F*	*F* or *M*

This learning rule applies only to the selected feature (*E*, environment; *L*, lever; *M*, magazine) in a given state transition, except in the case of food, *F*, which is constantly equal to 1, the value of the reward. The initial formulation in Lesaint et al. (2014) made the hypothesis that the value of *F* also had to be learned but this leads to an artefact in theoretical dopaminergic activity (see Fig. 7C of the original paper (Lesaint et al. 2014)): when looking at RPEs in the final state, when the animal receives the reward, instead of decreasing from an initially high value in the very first session, RPEs in the model start low and peak in session 2 before decreasing, as initially the value of food is also small compared with that of the previous feature. By fixing the value of *F*, this artefact disappears. This could mean that the value of the sensory features of the food reward do not need to be learnt, possibly because the animal has already encountered similar food pellets and their value is already learnt, or that it is learnt almost immediately with a different and much higher learning rate than what is used for the other features, an explanation which is reminiscent of the hypothesis by Rescorla and Wagner (1972) that the learning rate is dependent on the nature and saliency of the stimulus. In addition, because the animal is likely to visit the magazine and explore the environment during the ITI, the values of these two features are revised between states 7 and 0:

3 $$ \begin{array}{@{}rcl@{}} &&V(M) \leftarrow (1-u_{ITI}) \times V(M)\\ &&V(E) \leftarrow (1-u_{ITI}) \times V(E) \end{array} $$

with *u*_*ITI*_ the ITI update factor. Crucially, the level of this down-revision is dependent on the duration of the ITI, the longer it is, the more opportunities the animal has to revise these values, which is why we modelled different ITI durations by using a small (0.01) and large (0.1) value of *u*_*ITI*_. Whether the animal does indeed visit the magazine more often if the ITI is longer can be answered by looking at figure 3 in Lee et al. (2018) where they plot the correlation between dopaminergic activity and the number of visits to the magazine during the ITI. Importantly for us, they do this for a short and long ITI condition, and looking at the scatterplots, it seems to be the case, although we haven’t verified it statistically, that the number of visits is smaller in the short ITI than in the long ITI scenario (for instance the maximum number of visits to the magazine is around 20 versus 60 for short and long ITI conditions respectively).

Meanwhile, the MB system relies on learned transition *T* and reward *R* functions. The transition function aims at determining the probability of going from one state to the next given a certain action. It is updated after each state transition in the following manner:

4 $$ T(s_{t}, a_{t}, s_{t+1}) \leftarrow (1-\alpha) \times T(s_{t}, a_{t}, s_{t+1}) + \alpha  $$

with initial values of *T* set to 0 for all possible state and action combinations. Given that the environment is deterministic, *T* should converge perfectly towards values of 1 for all possible state transitions and stay at 0 for impossible state transitions, thus providing a perfect knowledge of the structure of the MDP. Similarly, the reward function is updated as follows:

5 $$ R(s_{t}, a_{t}) \leftarrow R(s_{t}, a_{t}) + \alpha (r - R(s_{t}, a_{t}))  $$

with *r* equal to 1 for (*s*_7_, eat) and 0 otherwise. Initially, R is equal to 0 for all state-action pairs. In this way, over time, R will also converge perfectly to a value of 1 for the (*s*_7_, eat) state-action pair. Using the current estimate of these functions, the agent can then compute an action-value function for each possible action *a*_*i*_ in the current state *s*_*t*_:

6 $$ Q(s_{t}, a_{i}) = R(s_{t}, a_{i}) + \gamma \sum\limits_{j}(T(s_{t},a_{i},s_{j}) \max_{k}(Q(s_{j},a_{k})))  $$

Finally, these *Q*-values are compared with each other so as to compute the Advantage function *A*:

7 $$ A(s_{t},a_{i}) = Q(s_{t},a_{i}) - \max_{j} Q(s_{t},a_{j})  $$

Once the FMF and MB systems have outputted the feature values and the advantages of the possible actions, these are integrated through a weighted sum:

8 $$ P(s_{t},a_{i}) = (1-\omega)A(s_{t},a_{i})+\omega V(f(s_{t},a_{i}))  $$

with *ω* bounded between 0 and 1, the weighting parameter favouring the FMF sub-system. These integrated values are then plugged into a softmax function to compute the probability of selecting each action:

9 $$ p(a_{t}=a_{i}) = \frac {e^{\frac{P(s_{t},a_{i})}{\tau}}}{{\sum}_{j}e^{\frac{P(s_{t}, a_{j})}{\tau}}}  $$

with *τ* the temperature parameter controlling the level of random exploration: when *τ* is large, the animal tends to disregard the difference in value of the different actions and chooses each action equiprobably, a behaviour known as random exploration, while if *τ* is small the difference between values is exacerbated leading to exploitation of what seems to be the most rewarding action.

In total this model has five parameters: the learning rate *α*, the discounting factor *γ*, the softmax temperature *τ*, the ITI update factor *u*_*ITI*_, and the integration factor *ω*. Note that the learning rate and discounting factor are shared by the MB and FMF systems despite the fact they could very well be different in reality. Originally, this was done to simplify the optimization of this model on experimental data (Lesaint et al. 2014) by reducing the number of parameters, and we are tributary to this original convention. Furthermore, the chosen values of *α*, *γ*, and *τ* are also derived from these original optimisations (Lesaint et al. 2015; Lesaint et al. 2014) and are fixed throughout this article while two possible values of *u*_*ITI*_ were hand-tuned to best illustrate how a long or short ITI might affect the model; as for *ω*, the value of this parameter was manipulated in different ways according to the specific aim of each section of this article. We refer the reader to Table 2 for the exact parameter values that were used. A more rigourous approach would of course have been to reoptimise the parameters on an experimental dataset such as that of Lee et al. (2018) which we attempt to simulate, but we did not have access to the precise numerical dataset which would have been necessary for such an undertaking and adopted a qualitative approach instead. As a counterpoint to this legitimate criticism, the robustness of these parameter values has been demonstrated by the fact that more rigourous optimisations were undertaken in two different studies using different methodologies in Lesaint et al. (2014) and Lesaint et al. (2015) and yielded quite similar values.

**Table 2:** Parameter values of the model were either directly from previous work by Lesaint et al. (2014) or hand-tuned, notably in the case of *u*_*ITI*_ to obtain illustrative effects; *ω* was either set to 1 or 0 for the FMF-only and MB-only models, or sampled from a probability distribution

	*α*	*γ*	*τ*	*u*_*ITI*_	r
Short ITI	0.03	0.8	0.15	0.01	1
Long ITI	0.03	0.8	0.15	0.1	1

Although the original work by Lesaint et al. (2014) and Lesaint et al. (2015) allowed sign-trackers and goal-trackers to have distinct sets of parameter values, we chose not to adopt this convention as our main interest is in illustrating how different levels of contributions of the FMF and MB systems can produce different behaviours independently of any other parameter effects.

## Predicted effects of ITI duration on the FMF-MB model

We first set out to verify the claim made in Lesaint et al. (2014) that by decreasing the ITI duration, goal-tracking would be favoured. This prediction, which was made without model simulations, is based on the fact that for the FMF system, the lever is preferable to the food cup because the latter is present during the ITI and subject to down-revision of value as the rat visits it without receiving a reward (see Eq. 3). Hence, if we reduce the duration of ITI and allow less time to experience unrewarded visits to the food cup, the learned value of the food cup should be higher and the preference for the lever should decrease. Importantly, this effect is entirely dependent on the FMF system.

**Fig. 3:**
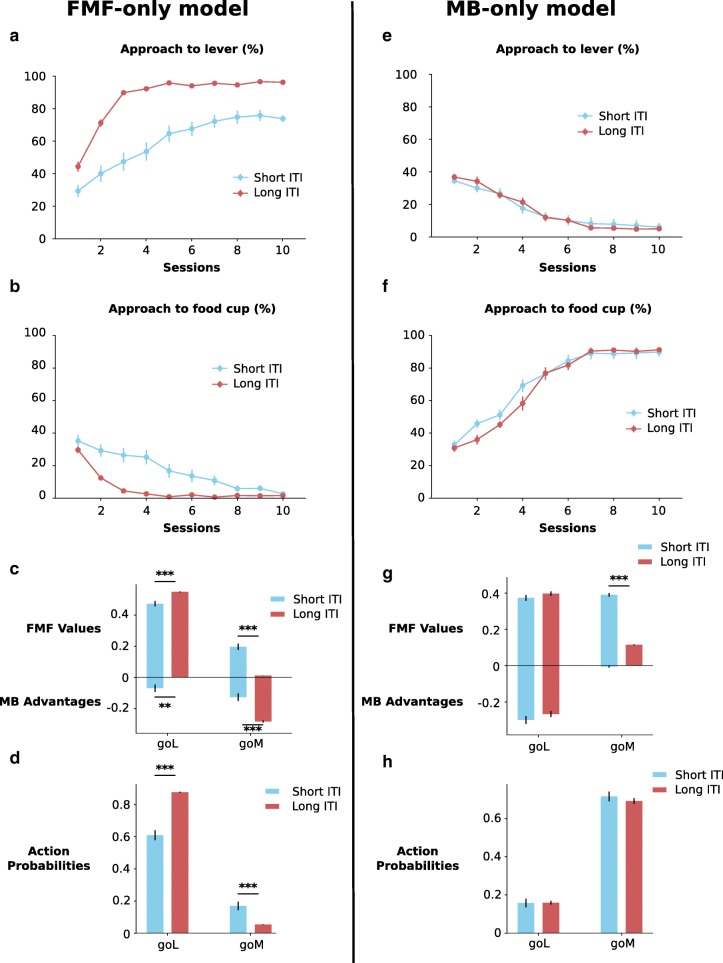
Behaviour of FMF-only (*ω* = 1, **a**–**d**) and MB-only (*ω* = 0, **e**–**h**) models. For each graph, we have plotted the mean ± s.e.m. **a** Approach to lever of simulations of the FMF-only model for different ITI durations. **b** Approach to the food cup of simulations of the FMF-only model for different ITI durations. **c** Effect of down-revision of food cup value on FMF and MB values of the FMF-only model. **d** Average softmax probabilities of engaging with either the lever or the food cup during the CS period of the FMF-only model for different ITI durations. **e** Approach to lever of simulations of the MB-only model for different ITI durations. **f** Approach to the food cup of the MB-only model for different ITI durations. **g** Effect of down-revision of food cup value on FMF and MB values of the MB-only model. **h** Average softmax probabilities of engaging with either the lever or the food cup during the CS period of the MB-only model for different ITI durations

To illustrate this, we present simulations of a model with only the FMF or the MB system being used to determine action by setting *ω* to either 1 (FMF system only) or 0 (MB system only) while keeping other parameters constant, and with two different levels of food cup revision during the ITI. In this way, only one sub-system is responsible for producing behaviour, but the second sub-system is still capable of learning from observation of events. Simulations were done for ten blocks of fifty trials in accordance with the experimental protocol of Flagel et al. (2009). After simulation, we plotted the average number of sign-tracking and goal-tracking choices in state 1, i.e. the number of times the agent selects goL or goM respectively, for each separate session (Fig. 3a, b, e and f). As predicted, when food cup value revision is low, as should be the case during short ITIs, the FMF-only model shows a definite increase in goal-tracking. If we decompose action probabilities into its FMF and MB contributions, we can verify that there is indeed an increase in the feature value of the food cup (Fig. 3c), and also an increase in the model-based advantage of going towards the magazine which is probably due to the fact that calculation of the transition function is biased by the animal’s choices and if the animal preferentially sign-tracks, the estimated probability of the transition from state 1 to state 3 will have less trials to converge towards 1. These differences straightforwardly impact the downstream outputs of the softmax function (Fig. 3d). In the case of the MB-only model, things are much simpler with little or no behavioural effect of down-revision of food cup value on either type of behaviour (Fig. 3e and f), despite the fact that there is a change in the FMF value function (Fig. 3g). In parallel, the advantage function remains unchanged. It is an interesting and unanticipated aspect of this model that the FMF system, by constantly biasing action towards the lever in the FMF-only model and thus affecting how much calculation of the transition function has converged, can have an impact on the MB system while the contrary does not happen for the MB-only model. This highlights how the two systems are complementary and how the dominant system may affect calculations of the second system. From these simple simulations, we can conclude that the initial claim is indeed correct: decreasing food cup value down-revision by shortening ITI duration should favour goal-tracking. Importantly, this prediction has been verified by Lee et al. (2018) as we shall see in more detail later. However, it is important to point out that this effect is dependent on the FMF system only: an agent using only MB learning will not show this effect, and as such it does not constitute hard evidence for the combination of FMF and MB learning.

If we look at our simulations from a slightly different angle, by taking short ITIs as our reference before increasing their duration, and suppose that we are dealing with a mixed population of goal-trackers and sign-trackers, a striking asymmetry emerges. As we have just explained, individuals with a dominant FMF system can nonetheless display goal-tracking behaviour if the ITI is sufficiently shortened to the point that the feature value of the magazine is hardly devalued during the ITI. In this way, it might be possible to obtain a population of purely goal-tracking individuals, despite the fact that underlying individual differences remain. On the other hand, since individuals with a dominant model-based system are largely insensitive to ITI duration according to this model (Fig. 3e and f), increasing the ITI will never result in a population of purely sign-tracking individuals but only allow what we might call “latent” sign-trackers, i.e. FMF-dominant individuals, to reveal themselves. In other words, this predicts that a more or less significant portion of the population should goal-track no matter how long the ITI lasts. Note that in the original STGT model aimed at accounting for the experimental results of Flagel and colleagues (Flagel et al. 2011), there were no pure MB agents, but simulated goal-trackers had a very small parameter *ω* < 0.05, which means that the FMF system was contributing to less than 5% of their behaviour (Lesaint et al. 2014; Lesaint et al. 2015) and that any impact on this system should be small. If the model is correct, such individuals might prove themselves to be practically insensitive to such an ITI manipulation. Interestingly, while the study by Lee et al. (2018) shows that at least a few individuals can be categorized as goal-trackers in the long ITI condition, at the level of the behaviour of the population the average goal-tracking behaviour seems overall quite weak compared with sign-tracking behaviour. Moreover, the percentage of contacts with the food cup during the CS period (i.e. goal-tracking behaviour) appears to progressively decrease sessions after sessions in the long ITI condition (Lee et al. 2018). In computational terms, this could either mean that there are too few animals using a predominantly model-based strategy or that the influence of the MB system can be unstable in time (similarly to its progressive decrease of influence on behaviour over training in favour of the development of habits in the instrumental paradigm (Daw et al. 2005)). We come back to this discussion later.

## Replication of main behavioural results from Lee et al. (2018)

**Fig. 4:**
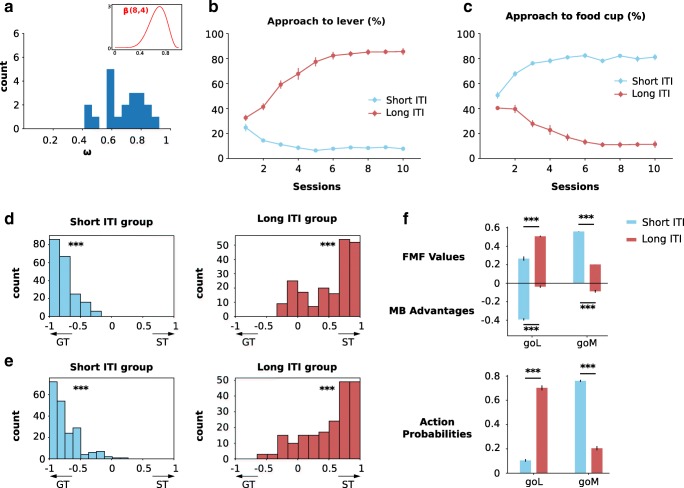
Simulations of the behaviour of a population with random *ω* parameter values. **a** Distribution of the *ω* parameters sampled from a *β* distribution which were then used for the simulations. The same values of *ω* were used in both short and long ITI condition. Inset: probability density function of the original distribution is biased towards 1 in accordance with the reported prevalence of sign-trackers. **b** Approach to the lever for different ITI durations (mean ± s.e.m.). **c** Approach to the food cup for different ITI durations (mean ± s.e.m.). **d** Distribution of differences in softmax probability of approach to lever and magazine for the two ITI durations. There is a significant bias towards goal-tracking choices in the short ITI group and a significant bias towards sign-tracking choices in the long ITI group. **e** Distribution of differences in average simulated number of approaches to lever and magazine for the two ITI durations. As expected from the differences in softmax probabilities, there is a significantly higher number of goal-tracking than sign-tracking trials in the short ITI condition and vice-versa in the long ITI condition. **f** Top: Effect of down-revision of food cup value during ITI of different durations on average FMF-values and MB action advantages. Bottom: Average softmax probabilities of engaging with either the lever of the food cup during the CS period for different ITI durations

In reality, individuals are unlikely to be purely FMF or MB, which is why, when aiming to replicate the results of Lee et al. (2018), we performed simulations with a population of twenty individuals with random *ω* values sampled from a biased *β* distribution (Fig. 4a) aimed at reflecting the fact that sign-trackers are supposedly more prevalent (Derman et al. 2018), although see the study by Morrison et al. (2015) for a case where the majority of individuals are goal-trackers. Once sampled, the same values of *ω* were used in both ITI conditions so as to perfectly isolate any effect of this factor. Similar results from an alternative simulation in which *ω* values were sampled from a uniform distribution are presented in the Supplementary Figure 1. In the long ITI condition, simulated rats increasingly approach the lever and avoid the magazine, while the opposite holds for the short ITI group (Fig. 4b and c). Because we kept the same parameters in the long and short ITI conditions, we could apply a repeated-measures ANOVA with two within factors corresponding to sessions and ITI conditions (instead of a mixed-design ANOVA with ITI as a between factor) on the proportion of trials where the simulated animal approached the lever and the magazine. We found very significant effects of sessions, ITI condition and of their interaction on both proportions (*p* > 0.0001). Post hoc *t* tests comparing the number of trials where the lever was chosen in each session produced significant differences for every session except the very first (smallest mean difference = 6.75 ± 0.84, *p* < 0.0001). Conversely, the number of times the magazine was approached was significantly higher in the short ITI group for every session, including the first (smallest mean difference = 2.55 ± 0.82, *p* < 0.0059). In summary, shortening the ITI produces an increase in goal-tracking choices in a mixed population of goal- and sign-trackers.

Lee et al. (2018) found similar results but preferred to report them through the distributions of behavioural session scores which included the response bias, the difference in probability of approach to the lever and the food cup, a latency of response index, and a Pavlovian Conditioning Approach (PCA) score (Meyer et al. 2012) which consists in the average of the three other scores. These different indices are designed to range between − 1 and 1 such that sign-tracking behaviour corresponds to scores closer to 1 and vice-versa for goal-tracking. These indices also allow for the possibility that an animal will interact repeatedly with one or both of the stimuli within a same trial. Using these metrics, the experimenters found a significant increase in the tendency to sign-track when comparing all four score distributions of the short and long ITI groups. Unfortunately, the original model (Lesaint et al. 2014) allows only for a single interaction with only one of the stimuli during the CS period of a trial, and does not attempt to model the latencies of responses either. This means that the only index at our disposal is the normalized difference in the probability of approach (*P*(go to Lever) − *P*(go to Magazine)) / (*P*(go to Lever) + *P*(go to Magazine)); the normalization was necessary given that the model has the possibility to explore instead of interacting with either cue. Given that the model provides direct access to the probability of each action at each trial through the softmax function, we used these probabilities (Fig. 4d) as well as the frequency of different choices made in the simulations (Fig. 4e), which is closer to the experimental methodology. The distributions of these two scores are significantly biased towards goal-tracking for the short ITI group (Wilcoxon’s signed-rank test: *μ* < − 0.74, *p* < 0.0001); to the contrary, the distributions corresponding to the long ITI group are significantly biased towards sign-tracking (Wilcoxon’s signed-rank test: *μ* > 0.51, *p* < 0.0001). Direct comparison of the short and long ITI groups also produced significant differences for both scores (Wilcoxon’s signed-rank test long minus short ITI: *μ* > 1.26, *p* < 0.0001). If we look for the origin of this effect by examining the average feature values and advantages (Fig. 4f top), we see that shortening the ITI has a significant positive effect on both the feature value and the advantage of going towards the magazine (Welch’s *t* test: *p* < 0.0001) and conversely a significant negative effect on the feature value and advantage of going towards the lever (Welch’s *t* test, *p* < 0.0001). These effects straightforwardly translate into a significant increase in the probability of going towards the magazine (Fig. 4f bottom, Welch’s *t* test: *t*(27.6) = 28.12, *p* < 0.0001) and of avoiding the lever (Welch’s *t* test: *t*(27.7) = − 25.44, *p* < 0.0001). In conclusion, shortening the ITI leads to an increase in both magazine value and advantage of going towards the magazine which both result in promoting goal-tracking, as found experimentally (Lee et al. 2018).

## Replication of dopaminergic patterns found by Lee et al. (2018)

**Fig. 5:**
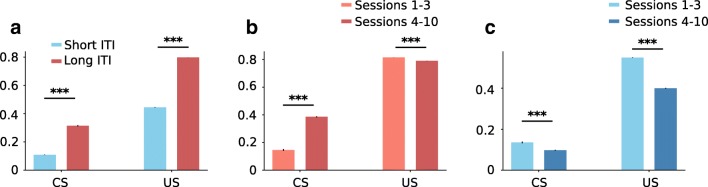
Reward prediction errors of the model at CS and US presentation for short and long ITIs **a** Reward prediction errors averaged across all sessions **b** Reward prediction errors for the long ITI simulations averaged in early and late sessions. **c** Reward prediction errors for the short ITI simulations averaged in early and late sessions

The study by Lee et al. (2018) also included measurements of dopamine release in the Nucleus Accumbens core (NAc) using fast scan cyclic voltammetry. These measurements found bursts of dopaminergic activity hypothesized to represent the RPEs computed by the FMF learning system. A first result is that, after averaging over all sessions, dopamine release at the time of the CS was significantly greater for the long ITI group than for the short ITI group. This result had not been explored by previous model simulations, but it is in fact predicted by the model (Fig. 5a, Welch’s *t* test: *t*(25.68) = − 43.36, *p* < 0.0001). Indeed, the RPE computed by the model at CS presentation is the RPE corresponding to transition from state 0 to state 1:

10 $$ \delta_{0} = \gamma \times max(V(M), V(L)) - V(E)  $$

To understand why this RPE should be bigger in a long ITI condition, it should be noted that, although it was only implied in the original paper by Lesaint et al. (2014) but apparently not known in Lee et al. (2018), the value of the environment *V* (*E*) is also revised during the ITI (see Eq. 3), meaning that it is smaller after a long ITI than after a short ITI, and the resulting RPE is greater. This increased activity simply reflects the fact that in a setting where rewards are less frequent, signals that predict rewards will cause more positive surprise.

On the other hand, the authors report that dopamine activity at US delivery is greater in the long ITI group than in the short ITI group and they expect this to be predicted by the model on the basis that this activity would correspond to the discrepancy between the reward and the value of the magazine which is smaller for the long ITI group. However, the original MDP is designed in such a way (Lesaint et al. 2014) that this discrepancy is not necessarily calculated on each trial because the value the reward is compared to is not always that of the food cup. Indeed, as shown in Fig. 1a, if the animal followed a sign-tracking strategy, then the RPE between states 2 and 5 which corresponds to the arrival of the US is:

11 $$ \delta_{2}=\gamma \times V(F) - V(L) $$

because the model hypothesizes that when the rat goes to the magazine in state 5, the feature it is comparing the reward to is the one it was previously focusing on, i.e. the lever and not the food cup (see Table 1). It might be tempting to propose an alternative feature function in which the animal focuses on the magazine when going from state 5 to 7, but this would make the value of the lever dependent on the value of the magazine rather than the food, meaning that increased ITI duration would affect both lever and magazine values. Alternatively, we could say that the RPE at US delivery should be measured when the reward is actually eaten, that is the RPE generated between states 7 and 0. However, in this case the RPE is:

12 $$ \delta_{7}=r-V(F)=0 $$

For this reason, we applied a simple correction to the feature function (Table 1), which we followed throughout the rest of this article, by supposing that the feature the animal focuses on when it eats is not the food but the food cup. In this case, we do indeed have an RPE equal to the difference between reward and value of the magazine at the cost of losing “real-world” significance of the action-feature function. However, a significant advantage of this new formulation is that it allows the updating of the food cup value even in trials during which the animal sign-tracked. Indeed, under the previous conventions, if the animal sign-tracked it would never perform an action focused on the food cup feature and the corresponding value would remain the same despite the fact that the reward really is retrieved from the food cup. As a consequence, there is a slightly increased tendency to goal-track using this convention compared with the previous version of the model (comparison not shown here). Using this new model convention, RPEs at the US are significantly greater in the long ITI group than in the short ITI group (Fig. 5a, Welch’s *t* test: *t*(21.80) = − 252.87, *p* < 0.0001), which is consistent with Lee et al. (2018).

As learning progresses and the feature values gradually converge, the RPEs at CS and US appearance times should evolve between early and late sessions. Lee et al. (2018) investigated this by comparing dopamine activity between the first three sessions and the last seven. They report that in the long ITI group, dopamine release to lever presentation was significantly greater in late sessions than in early sessions, reflecting the increase in lever value, while dopamine activity at reward delivery remains important due to the positive surprise of receiving reward from a low-value magazine. In simulations of the long ITI group (Fig. 5b), there is indeed a very significant increase in average RPE value between early and late sessions at CS presentation (paired *t* test: *t*(19) = − 56.20, *p* < 0.0001) which corresponds to the difference *V* (*L*) − *V* (*E*) which increases as *V* (*L*) does, but also a very significant decrease at US delivery (paired *t* test: *t*(19) = 65.86, *p* < 0.0001). Although highly significant, it should be noted that the magnitude of this decrease is visibly quite modest(Fig. 5b) which might contribute to explaining why it is not detected experimentally. Additionally, there is little variability between rats in the simulations (as evidenced from the standard errors of the mean in Fig. 5) probably because they share the same parameters except for *ω*, which increases the likelihood of detecting such small effects. As previously explained, the RPE at US presentation is equal to *V* (*F*) − *V* (*M*), and the slight decrease of this difference is probably a sign that, through rare occasions where the magazine is indeed selected in state 1, its value does manage to increase slightly. Another potential explanation for the discrepancy with the experimental data is the presence in the animals of a process of forgetting, such as the one used in Ito and Doya (2009) or Cinotti et al. (2019) in which an unused action (in this case it would be a feature) sees its value decrease. Such a mechanism would ensure that the magazine does not slowly and incrementally accumulate value for each time it is selected.

Concerning the short ITI group, the experimental results (Lee et al. 2018) showed that dopamine release during the CS period did not significantly change, while there was a significant decrease during the US period. Similarly in our simulations, we find a significant decrease in RPEs during the US period (paired *t* test: *t*(19) = 78.55, *p* < 0.0001) reflecting the fact that a shorter ITI means that the value of the magazine can converge towards a higher value and thus *V* (*F*) − *V* (*M*) decreases more strongly. However, there is also a small but significant decrease at CS presentation (paired *t* test: *t*(19) = 9.66, *p* < 0.0001) which is absent in experimental data and which we find difficult to explain. We can suppose that the RPE at this point corresponds to *V* (*M*) − *V* (*E*) as short ITIs enhance goal-tracking at the expense of sign-tracking. Thus, the decrease of this RPE could either be due to a faster decrease of *V* (*M*) compared with *V* (*E*) which is unlikely as both values should increase with learning until they converge (see Supplementary Figure 2 for a more direct visualization of RPE evolution between sessions), or to a faster increase of *V* (*E*). Maybe, the value of the magazine reaches its maximum value in early sessions and then evolves very slowly, while that of the environment reaches its maximum in later sessions as the environment value is increased less frequently.

It is interesting to note that the simulations point to a reversal in DA activity at CS time between short and long ITI groups, with rats in the long ITI group seeing a significant increase and rats in the short ITI group a significant decrease, an effect that could perhaps be verified experimentally with a greater number of subjects. Because RPEs at this point correspond to the difference between most valuable feature and the environment (see Eq. 10), this effect is certainly due to the fact that the value of the environment is greater in the short ITI group, as previously explained.

## Detailed analysis of behaviour during the CS presentation window

**Fig. 6:**
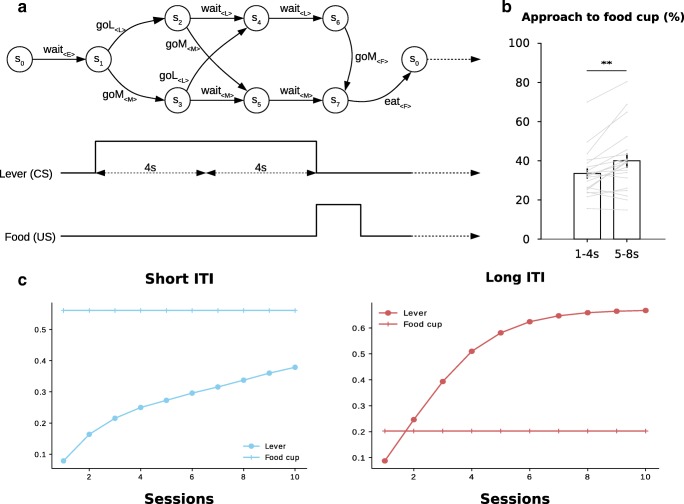
Simulations of the original model with added intermediate steps within the CS presentation period. **a** New task structure with added intermediate steps and possible transitions from the lever to the food cup and vice-versa in states 2 and 3. In addition, the possibility of exploring the environment was deleted for simplicity. **b** Probability of approach to the food cup during the first and last four seconds of the CS presentation period. Bar plot represents mean probability and grey lines individual probabilities. **c** Average feature values of the lever and magazine in the short and long ITI conditions across sessions. In the long ITI group, the value of the less favourable feature, which is the food cup, is stagnant, while in the short ITI the value of the lever keeps increasing, causing possible ambiguity which could explain unstable behaviour during the CS period

Lee et al. (2018) also published a result which cannot be accounted for by the original version of the FMF-MB model as presented in Lesaint et al. (2014). Indeed, this model was designed to account for a single behavioural response per CS period of a trial. In contrast, in late sessions, Lee et al. (2018) found that when the 8 s CS period is divided in two, while in the long ITI group sign-tracking dominates throughout the entire CS period, in the short ITI group after learning, goal-tracking is limited to the first 4 s of the CS period, while sign-tracking takes over in the last 4 s. This result is at first glance very surprising as it seems to stand in contradiction with previous reports that food cup CRs are usually concentrated in the last seconds of CS presentation (Holland 1977; Nasser et al. 2015). It also stands in contradiction with what we would expect from the original model. Indeed, if goal-trackers are predominantly using a MB strategy then, if we allow the model to make a second decision during the CS period (Fig. 6a), it is plausible that the simulated goal-trackers shift from sign-tracking during early CS period to goal-tracking during late CS period, as they search for the shortest possible route to the reward. The reverse on the other hand undermines the MB strategy by adding an intermediate step between sign-tracking and reward consumption. To prove this, we ran simulations of the original model with additional intermediate steps within the CS period (Fig. 6a) and found a significant increase in goal-tracking in the last 4 s compared with the first 4 s (Fig. 6b; Wilcoxon signed-rank test: *W* = 21.5, *p* = 0.0018).

A plausible explanation of the result by Lee et al. (2018) might be found in the relative feature values of the lever and the magazine. Indeed, if we turn back to the original single-action model and plot the average FMF value of the magazine and the lever we find that in the case of the long ITI, both values stabilize and stay far apart, while in the short ITI group, the value of the lever keeps increasing despite not being selected very often (Fig. 6c). The stability for the long ITI group could be viewed as an absence of uncertainty about estimated feature values, and thus a good reason to simply select one feature to focus on during the CS period: the lever. This is consistent with the stable sign-tracking behaviour observed after learning throughout the entire CS period in the long ITI group in Lee et al. (2018). In contrast, the instability of the lever value for the short ITI group might cause a growing uncertainty as to which feature to focus on. The fact that the value of the magazine remains superior might be the reason why the animals first visit it. Then, after visiting the magazine, rather than waiting for the end of the CS period, the rats might be attracted by the lever because of the high uncertainty associated to its estimated value, which could be viewed as a form of directed exploration (Daw et al. 2006; Wilson et al. 2014). This might also explain some reports that goal-tracking is an unstable phenotype (see figure 2 in Nasser et al. (2015) and figure 3 in Meyer et al. (2012) in which a population initially displaying mostly goal-tracking behaviour gradually breaks up into two goal-tracking and sign-tracking sub-populations). Indeed, looking at the curves, it seems very likely that the feature value of the lever will eventually catch up with that of the magazine, as shown in simulations with an extended number of sessions in Supplementary Figure 3. This would predict that extending the number of acquisition sessions could enable a slow learning process to be revealed, which could lead some rats to ultimately sign-track. Consistent with this interpretation, some reports show some shifting in the distribution of responding from goal- to sign-tracking even across just 5 days of acquisition (Meyer et al. 2012).

## Discussion

In this paper, we presented novel simulations of a sign-tracking/goal-tracking (STGT) model first proposed by Lesaint et al. (2014). The goal was to assess whether the model could account for all behavioural and neurophysiological results reported in the recent paper by Lee et al. (2018). This latter study was itself designed to test some specific predictions of the STGT model, namely that manipulating the duration of the inter-trial interval (ITI) so that animals have either more or less time to visit the unrewarded magazine during that period would change the relative proportions of sign- versus goal-tracking behaviour in the population, as well as the dopamine response pattern. More precisely, the STGT model assumes a down-revision of magazine value each time it is visited but unrewarded during the ITI, which constitutes a possible explanation for why the dopamine response at CS in goal-trackers does not reflect an increase in reward expectancy (Flagel et al. 2011). This led the model to predict that shortening the duration during which animals could visit the unrewarded magazine during the ITI (or simply making the magazine inaccessible during ITI) would lead to an increase of goal-tracking behaviours as well as a restoration of a reward prediction error-like dopamine response pattern (Lesaint et al. 2015). Lee et al. (2018) tested two different ITI durations and found that increasing this duration led to increased sign-tracking and increased dopamine phasic response to the US. While these results are consistent with the model predictions, the same researchers also found behavioural and neural responses which had not been explored by previous modelling work and which we addressed here.

Firstly, after averaging over all sessions, Lee et al. (2018) found that dopamine release at the time of the CS was significantly greater for the long inter-trial group than for the short inter-trial group. This result had not been explored by previous model simulations, but we showed here through novel simulations that it is consistent with the STGT model and simply corresponds to a greater valuation of rewards and reward-predicting stimuli in a setting with less frequent rewards. Secondly, the same authors report that dopamine activity at US delivery is greater in the long ITI group than in the short ITI group, a fact which cannot be accounted for by the original formulation of the STGT model. Here we showed that the increase of dopamine activity at US delivery can be accounted for by extending the STGT model by supposing that the feature the animal focuses on when it eats is not the food but the food cup. Thirdly, after an initial learning period, the authors report a stable sign-tracking behaviour throughout the entire CS period in the long ITI condition, as opposed to initial goal-tracking during the first 4 s of the CS period followed by later sign-tracking in the short ITI condition. This last result goes beyond the original STGT model which had been designed to account for a single behavioural response (Lesaint et al. 2014). Nonetheless, using this model, we showed that the uncertainty associated to learned feature values in the model is reduced after learning in the long ITI condition, while the uncertainty associated to the lever remains high in the short ITI condition due to the continuous increase in its value. The former case would be consistent with the stability of sign-tracking behaviour in the long ITI condition while the latter could provide an explanation why animals in the short ITI condition are first attracted to the food cup (which has the highest value in the model) and then by the lever (which has the highest uncertainty in the model). The model further predicts that extending the number of experimental sessions would eventually lead goal-tracking behaviour to progressively diminish in both conditions.

These results have important implications for the understanding of the possible neural mechanisms underlying individual differences in Pavlovian autoshaping. They further confirm the computational interpretation that sign-trackers may rely more on model-free (MF) learning processes while goal-trackers may rely more on model-based (MB) learning processes (Lesaint et al. 2014; Dayan and Berridge 2014). They bring detailed analyses of the respective contributions of MB and MF learning mechanisms which may explain specific results of Lee and colleagues (2018), while remaining consistent with experimental results previously accounted for by the model (Robinson and Flagel 2009; Flagel et al. 2011; Saunders and Robinson 2012). They moreover lead to testable predictions, which could lead to future experiments aiming at further assessing the present computational hypotheses. In particular, the model predicts that animals whose learning process can be modelled as pure MF should be sensitive to down-revision of food cup value through both increase and decrease of ITI duration. In contrast, animals whose learning process can be modelled as pure MB should be insensitive to this manipulation of the protocol so that a population consisting of a mix of FMF- and MB-dominant individuals could be transformed entirely into a goal-tracking population under short ITI conditions but not into a purely sign-tracking one if ITI durations are increased indefinitely.

A recent article by Derman et al. (2018) challenges the idea that sign-tracking and goal-tracking are supported by distinct neurological processes. More precisely, this paper reports that, contrary to the conclusions of Morrison et al. (2015), sign-tracking behaviour is in fact sensitive to outcome devaluation. This could be an argument against our model, because model-free learning systems have become equated with habitual, i.e. insensitive to devaluation, behaviour since the seminal article of Daw et al. (2005) which proposed a model of the development of habits as a transition from model-based processes to model-free processes. However, the work of Derman et al. (2018) suffers from the notable absence of goal-tracking individuals for comparison. Indeed, the authors are able to find that sign-tracking individuals are sensitive to outcome devaluation but without a comparison with goal-tracking individuals, we do not know whether this sensitivity is greater or smaller than for goal-tracking individuals. Furthermore, the equivalence between model-free and habitual behaviour should probably be questioned. As the model-free system continuously learns from observed outcomes, it is in fact perfectly capable of readjusting its behaviour after outcome devaluation. The only issue is that it will do so much more slowly than a model-based system in a complex environment with many states which each have their own value which needs to be recomputed, as is the case in the study of Daw et al. (2005). In a simple Pavlovian task, there is no reason to believe that a model-free system will not be able to adjust in response to outcome devaluation. A second important result from Derman et al. (2018) which poses a challenge to a dual-process theory of sign- vs. goal-tracking behaviour is that, if rat subjects develop a goal-tracking strategy in response to an auditory CS, this strategy will then block the development of a sign-tracking strategy for a lever CS. According to the authors, this means that goal and sign-tracking strategies rely on a common prediction error learning process. This result stands in contradiction with the pharmacological experiments of Flagel et al. (2011) which shows that dopaminergic inhibition prevents the acquisition of sign-tracking behaviour but not of goal-tracking behaviour. Secondly, as Derman et al. were unable to generate a sub-population of goal-trackers in the other experiments presented in that same paper, we are unsure as to whether the goal-tracking behaviour in response to the auditory tone is produced by a MB-dominant learning system or by a FMF-dominant system. In other words, the researchers might be dealing with animals which are what we might call “latent” sign-trackers, individuals with a dominant FMF system which nonetheless develop a goal-tracking strategy, in which case the blocking of one kind of behaviour by the other is indeed the sign of a dominant learning system. However, this would not prove that there are not other kinds of individuals with a more complex learning system.

The present work also highlights a characteristic of simulated ST and GT behaviours which had not been addressed by previous models (Lesaint et al. 2014; Kaveri and Nakahara 2014): the tendency to goal-track less and less along training. This result is of particular importance since several studies have reported that goal-tracking is an unstable phenotype (Derman et al. 2018; Nasser et al. 2015), which sometimes may eventually disappear as conditioning goes on. Some reports show some shifting in the distribution of responding from goal- to sign-tracking even across only 5 days of acquisition (Meyer et al. 2012). In contrast, Flagel and colleagues (Flagel et al. 2011) found that both sign-tracking and goal-tracking behaviours were stable and robust throughout sessions. Because the present computational model accounts for the experimental results of Flagel et al. (2011) while at the same time predicting a tendency to goal-track less along training, the model has the potential to reconcile these different studies by suggesting that small differences in protocol can lead to variability and instability in ST and GT behaviours. It suggests that the behavioural phenotype may be affected by not only the duration of training, but also precise timing between events of the task, duration of the ITI, and any manipulation which can affect the values and uncertainties associated to stimuli. Similarly to the apparent discrepancy between studies showing similar proportions of STs and GTs (Flagel et al. 2011), a prevalence of STs or in contrary a majority of GTs (Morrison et al. 2015), it is worth noting that the same computational model has been also applied to pigeon negative automaintenance paradigm (Lesaint et al. 2014) where it could explain the discrepancy between different studies showing different proportions of individuals either able (putatively model-based) or not (putatively model-free) to refrain from pecking a light when this action prevents the obtention of reward.

Interestingly, the progressive decrease in goal-tracking behaviour cannot here be directly linked to a relative change in influence of the model-based system over behaviour. The relative contribution of MB and MF modules to decision-making in the model is indeed determined by a parameter *ω* which is fixed in an individual and stable in time. In contrast, computational models for the coordination of MB and MF reinforcement learning tackling experimental data in the instrumental conditioning and navigation paradigms have commonly assumed a progressive shift from MB to MF across learning to explain the tendency of animals to develop behavioural habits (Daw et al. 2005; Keramati et al. 2011; Khamassi et al. 2013; Viejo et al. 2015; Dollé et al. 2018). In future work, it would be interesting to extend the present STGT model so that it progressively shifts control over decision-making from MB to MF, and study whether this can further expand the model’s explanatory power with respect to experimental data.

Finally, the present set of results have important implications for the understanding of the mechanisms underlying drug addiction. The present paradigm assessing individual differences in response to conditioned stimuli in a Pavlovian appetitive task had indeed initially been proposed as a model of differential susceptibility to drug addiction (Saunders and Robinson 2013). In this paradigm, the fact that some individuals (sign-trackers) develop a strong attraction towards stimuli that invariably predict the occurrence of a biologically relevant event such as a food reward, can be seen as a model for individuals that develop a strong attraction for stimuli that predict drug rewards. Sign-trackers have indeed been found to be more sensitive to drug-predicting stimuli than goal-trackers (Flagel et al. 2009). The fact that in the present model sign-trackers rely more on relatively inflexible model-free behaviours than on flexible model-based decisions could provide a computational basis for further understanding why individuals who become addicted are unable to shift their thoughts and actions away from drugs and drug-associated stimuli. Interestingly and consistently, sign-trackers are more prone to display an automatic behaviour towards reward-predicting cues, in the sense that the same animals may be less sensitive to extinction of conditioning (Ahrens et al. 2016) or to devaluation of the reward (Morrison et al. 2015; Nasser et al. 2015; Patitucci et al. 2016) than goal-trackers. The present model could thus add to a growing computational literature addressing drug addiction in terms of model-based and model-free learning mechanisms (Simon and Daw 2012).

## Electronic supplementary material

Below is the link to the electronic supplementary material.

(PDF 219 KB)
