# Supplementary material for: Impacts of inter-trial interval duration on a computational model of sign-tracking vs. goal-tracking behaviour
Source: Psychopharmacology (Berl). 2019 Jul 31;236(8):2373–88. doi: 10.1007/s00213-019-05323-y (PMC6695359; doi:10.1007/s00213-019-05323-y)
Supplement: Supplementary file 1 — (PDF 219 KB) [file 213_2019_5323_MOESM1_ESM.pdf]

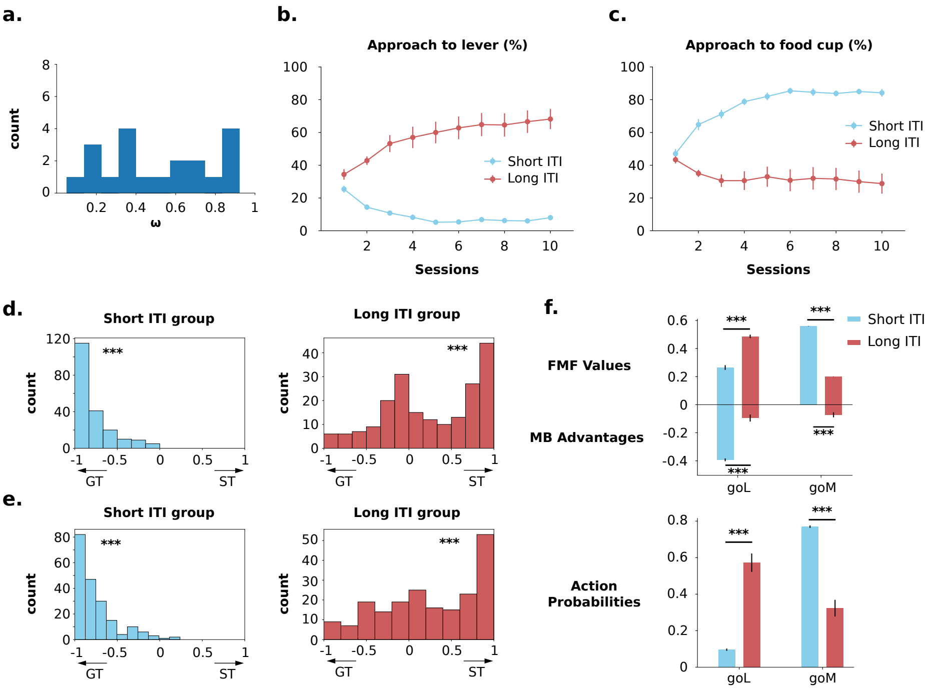

Fig. 1 Simulations of the behaviour of a population with random ω parameter values sampled from a uniform distribution. **(a)** Distribution of the ω parameters sampled from a uniform distribution over the range $[0, 1]$. **(b)** Approach to the lever for different ITI conditions. **(c)** Approach to the food cup for different ITI conditions. **(d)** Distribution of differences in probability of approach to lever and magazine for the two ITI conditions using the output of the softmax function. **(e)** Distribution of differences in average simulated number of approaches to lever and magazine for the two ITI conditions. (**f**) Top: Effect of food cup devaluation on FMF and MB values of the FMF model. Bottom: Average probabilities of engaging with either the lever of the food cup during the CS period for different devaluation levels.

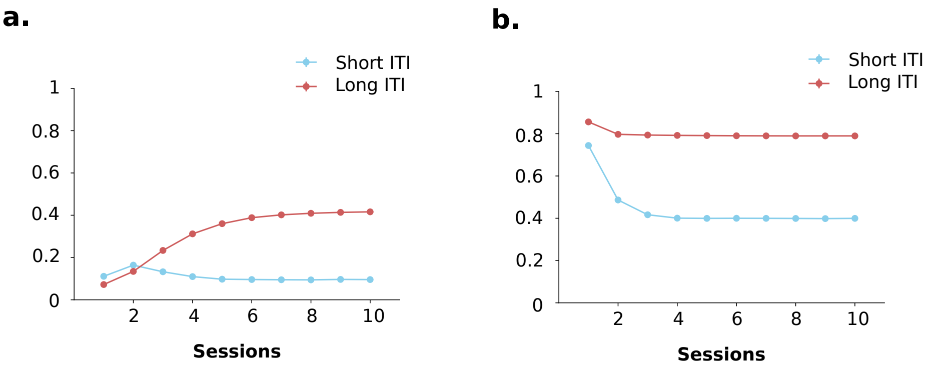

Fig. 2 Evolution of the RPEs of the model at CS and US presentation between sessions. Curves plot mean RPEs for each session, error bars corresponding to the standard error of the mean are too small to be seen. **(a)** Average RPEs at CS presentation show a gradual increase in the long ITI group as they learn the positive value of the lever, while RPEs in the short ITI group remain low. **(b)** Average RPEs at US delivery show little variation in the long ITI group and a strong one in the short ITI group. Notably, RPEs do not converge to 0, as is the case for DA experimentally, as the value of the magazine cannot converge to the value of the reward for computational reasons (see mathematical demonstration for this effect later in the **Supplementary Information**.)

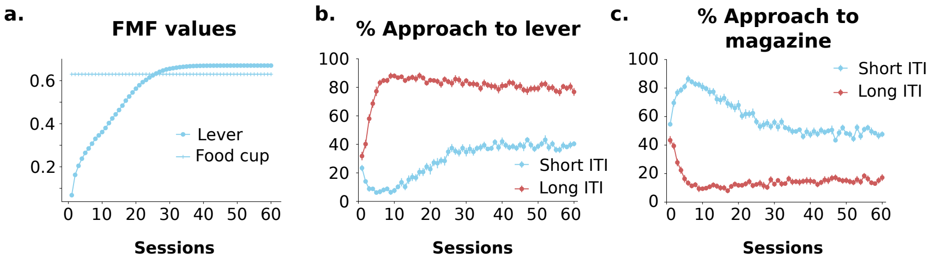

Fig. 3 Predicted effect of long-term effects of ITI manipulation on sign-and goal-tracking behaviour on the original model with a single action per CS period. In these simulations, having found that the FMF value of the lever seemingly keeps increasing, we continued simulations for a much greater number of sessions to see whether the FMF value of the lever would eventually catch up with that of the food-cup and the effect this would have on behaviour. **(a)** FMF values of the lever and food cup in the short ITI condition. Contrary to the long ITI condition (see main text and fig.6) where FMF values converge quite rapidly, the value of the lever keeps increasing and given enough time overtakes the value of the food cup which stays high. **(b)** In the short ITI condition, approach to the lever initially drops as the difference between magazine and lever values increases, but eventually should increase, contrary to what we observe in the long ITI condition where behaviour converges towards sign-tracking quite rapidly and stays stable. **(c.)** In parallel, the opposite should be true for the approach to the magazine, with an initially strong goal-tracking tendency in the short ITI condition progressively weakening, but remaining high nonetheless.

Demonstration that even in the absence of ITI value revision US RPEs cannot converge to 0:

We will first demonstrate that the value of the magazine from one trial to the next follows an arithmetico-geometric progression (”suite arithmético géométrique” in French), the limit of which we will then determine and prove to be different from the value of the food reward, even in the case where there is no ITI down-revision of the magazine value, thus resulting in a constant error signal. If we take a goal-tracking trial in which the animal progresses from state 0 to state 1, from state 1 to state 4, from state 4 to state 7 and finally from state 7 to state 0 of the next trial, then the value of the magazine is revised three times:

- from state 1 to state 4:

$$V_{s_4,t}(M) = (\gamma V_{s_1,t}(M) - V_{s_1,t}(M)) \times \alpha + V_{s_1,t}(M) = V_{s1,t}(M) \times (1 - \alpha + \alpha\gamma) \quad (1)$$

- from state 4 to state 7:

$$V_{s_7,t}(M) = V_{s4,t}(M) \times (1 - \alpha + \alpha\gamma) \quad (2)$$

- from state 7 to state 0 of the next trial:

$$V_{s_0,t+1}(M) = V_{s_7,t}(M) + \alpha \times (r - V_{s_7,t}(M)) \quad (3)$$

Combining these three equations we get: $V_{s_0,t+1} = (1-\alpha)(1-\alpha+\alpha\gamma)^2 \times V_{s_0,t} + \alpha \times r$ which is indeed an arithmetico-geometric progression. Because the absolute value of the term in front of $V_{s0,t}$ is inferior to 1, we are assured that this sequence converges, and that its limit will be $\frac{\alpha \times r}{1-(1-\alpha)(1-\alpha+\alpha\gamma)^2}$ which is different from r. Thus there will always be a persistent difference between the reward and the value of the magazine as illustrated in supplementary figure 2. This persistent difference would disappear if the discounting factor was set to 1, i.e. if no discounting takes place, as $\frac{\alpha}{1-(1-\alpha)(1-\alpha+\alpha\gamma)^2}$ would be equal to 1.

If we had taken the ITI into account, then we would have to include the following transition :

$$V_{s_0,t+1}(M) \leftarrow (1 - u_{ITI}) \times V_{s_0,t+1}(M) \quad (4)$$

and the value of the magazine would then converge to $1 - u_{ITI}$ which is closer to 1 as the ITI duration diminishes, in the absence of discounting ($\gamma = 1$).
